# Detection of physiological concentrations of GABA using dielectric spectroscopy - A pilot study

**DOI:** 10.2478/joeb-2023-0006

**Published:** 2023-12-31

**Authors:** Jie Hou, Runar Strand-Amundsen, Ørjan G. Martinsen

**Affiliations:** 1 Department of Physics, University of Oslo, 0316 Oslo, Norway; 2 Department of Clinical and Biomedical Engineering, Oslo University Hospital, 0372 Oslo, Norway

**Keywords:** Dielectric relaxation spectroscopy, GABA, gamma-aminobutyric acid, permittivity

## Abstract

Gamma-aminobutyric acid (GABA) is a major inhibitory neurotransmitter that is present at a relatively low level throughout the normal adult human brain. Abnormal GABA levels are found in people with neurodegenerative disorders such as Parkinson’s disease, epilepsy, schizophrenia, depression, and others. Being able to measure the GABA concentration would be beneficial for patient groups with fluctuating GABA levels for better diagnosis and treatment. In this study, we explore the feasibility of using dielectric relaxation spectroscopy for the detection of GABA concentrations within a physiological range, with the perspective of miniaturization and use during implantation. Utilizing machine learning techniques, we were able to differentiate GABA concentrations down to 5 μm. This work investigates a novel use of dielectric relaxation spectroscopy, to assess if physiological GABA concentrations can be detected through permittivity measurements.

## Introduction

To tackle the neurodegenerative disorders associated with gamma-aminobutyric acid (GABA) deficiency, it is imperative to advance the development of in vivo GABA detection techniques. Especially with technologies that potentially can be miniaturized, allowing for use during implantation. However, the dynamics of the GABA molecule are complex, with respect to the electrical, chemical and biological properties. GABA interacts with different GABA receptors through several different processes to transmit signals between neurons.

GABA-related abnormalities can impact one or more of these processes. This positions GABA at the core of a series of neurological conditions and diseases. A significant loss of GABA content was found in patients with Alzheimer’s disease in cerebellum (1.1 ± 0.3 μmol/g vs. 1.8 ± 0.5 μmol/g), in the temporal cortex (0.5 ± 0.2 μmol/g vs. 1.3 ± 0.8 μmol/g) and in the occipital cortex (0.8 ± 0.2 μmol/g vs. 1.4 ± 0.6 μmol/g) [1]. Moreover, synaptically released GABA may reach a transient concentration of 1.5 - 3.0 mM, whereas in the central nervous system extracellular space has concentrations ranging from 0.2 to 2.5 μM [2], [3]. At the present time there are no simple measuring techniques available to detect GABA levels in vivo, and the available approach is to use magnetic resonance spectroscopy (MRS) and high performance liquid chromatography (HPLC) [4]. Some other neurotransmitters, like dopamine, can be detected in vivo with well-established electrochemical methods like cyclic voltammetry or amperometry. The non-electroactive properties of the GABA molecule make it more dificult to detect, limiting the available methods for detection to invasive chemical and biological approaches. These approaches are often destructive. Since GABA is mainly found as a zwitterion (contains an equal number of positively and negatively charged groups), it has two charged groups that are well separated from each other, thus, making it a polar molecule with a dipole moment. The polarity of the GABA molecule indicates that dielectric relaxation spectroscopy (DRS) should be a proper method for measuring different GABA concentrations, as DRS is often used to investigate the motion of molecules that have an electric dipole moment.

Over the years, the dielectric properties of biological materials have been studied extensively, to understand the associations between these properties and various tissue states and pathologies [5]. For example, in medical diagnostics, the permittivity properties are essential in order to detect tumor cell nests [6]. Dielectric properties help us to understand the structure and dynamics of biological materials on the molecular level. Accurate knowledge of these properties are used in medical diagnostics, monitoring, and therapeutic technologies [7]. DRS is commonly used for non-destructive monitoring of dielectric properties of biological materials that undergo physical or chemical changes. DRS has been used to assess various problems related to agriculture, food, and biomedicine. Biological processes that involve changes in the concentration of substances that contain polar molecules, or biological processes that result in the formation of chemical species, can be studied using DRS [8].

To understand the practical potential of the DRS method with respect to detecting physiological GABA concentrations in vivo, it is important to understand the limitations related to the present non-invasive clinical approach for GABA detection; MRS. It is possible to measure the localized concentrations of GABA within the brain using proton MRS [9]. However, MRS machines present challenges due to their high cost and complexity. Most importantly, MRS is a technology that can presumably not be miniaturized to the level of implantation. Another common technique to measure GABA in microdialysates is HPLC coupled with fluorescence [4]. However, this method has a low temporal resolution as it requires a long time to collect enough samples to be measured. Compared to these methods, the DRS is based on cheap and readily available equipment, with small constraints to spacing compared to the MRS, making it considerably more cost-effective, fast, and a feasible choice for clinical use, given that it can be used to detect GABA levels. Furthermore, there is a potential for miniaturization with respect to the DRS technology, opening for the possibility of making compact implantable devices, akin to those employed in Deep Brain Stimulation (DBS) [10]. Such a device could be used to make frequent or even continuous measurements in patients, opening the possibilities for a much more dynamic and detailed monitoring of GABA concentrations in the brain and during different circumstances, than what is possible with MRS and HPLC. Our aim in this study is to investigate the feasibility of using DRS measurements for the determination of physiological concentrations of GABA in solvent water.

## Materials and methods

We used DRS to measure the permittivity of physiological GABA concentration solutions.

## Dielectric relaxation spectroscopy theory

Dielectric relaxation spectroscopy is a subcategory of impedance spectroscopy, where the dielectric properties of a material are measured as a function of the frequency of an applied electric field. Permittivity measurements imply measuring the total polarization of the material in a sample within the close proximity of the electrodes of the measuring probe. The unit of permittivity is farad per meter (F/m or F · m^−1^). The measured quantity in DRS is complex relative permittivity, mathematically expressed as:

1 $$\epsilon_{r}^{\prime }(\omega )=\frac{\epsilon^{\prime }(\omega )}{\epsilon_{0}}=\epsilon_{\text{r}}^{\prime }(\omega )-\text{j}\epsilon_{\text{r}}^{\prime \prime }(\omega )=\epsilon_{\text{r}}^{\prime }(\omega )-\text{j}\frac{\sigma (\omega )}{\omega \epsilon_{0}}$$

Complex relative permittivity is a function of the angular frequency ω, relative to the permittivity in vacuum, ϵ_0_. Here $\epsilon_{\text{r}}^{\prime }$ is the real part of the complex permittivity often called the “dielectric constant” or “relative permittivity”. This describes the properties of a material that affects the Coulomb force between two point charges in the material. $\epsilon_{\text{r}}^{\prime }$ describes the interaction between a dielectric material and an externally applied electric field, showing to what extent a material concentrates electric flux. Relative permittivity describes the ability of a biological material to store energy when an electric field is applied. $\epsilon_{r}^{\prime \prime }$ is the imaginary part of the complex permittivity, often called the “dielectric loss” or “loss factor”. It describes the energy absorption or attenuation of the material, reflecting the dissipative nature of the material. The dissipation or absorption of energy is partially converted to heat [7].

## GABA sample preparation

GABA (purity ≥ 99%) was purchased from Sigma-Aldrich, and used as received. To prepare accurate GABA sample concentrations for the experiment, quantities of GABA powder were calculated using the equation:

2 m=M⋅n=M⋅c⋅V

where m is the mass of the GABA powder in gram (g), M is the molar mass of GABA, M = 103.12 g/mol. c is the concentration of GABA with unit [mol/kg = molal = m] and V is the volume of solvent water, V = 0.5 L = 0.5 kg.^1^ The deionized water used as solvent water for all GABA concentrations was the “type II” water, which is defined as having a resistivity of >1 MΩ-cm, and a conductivity of <1 μS/cm. Based on the physiological concentrations of GABA in the human brain [1, 11, 2, 3], the following concentrations were used in this study: 5 μm, 10 μm, 20 μm, 50 μm, 100 μm, 200 μm.

## Instrumentation and DRS measurement setup

The permittivity data was measured with an R140 vector network analyzer (VNA) and a DAK-3.5 probe. The R140 VNA covers a frequency range of 85 MHz to 14 GHz and a frequency resolution of 25 Hz, and was developed by Copper Mountain Technologies (Indiana, USA). The DAK-3.5 probe, which was developed by Schmid & Partner Engineering AG (Zürich, Switzerland) covers a frequency range of 200 MHz to 20 GHz, and its inner and outer conductor dimensions were a = 0.93 mm and b = 3.5 mm, respectively. After each measurement, the relative dielectric constant, the dielectric loss, and the conductivity of the material under test were calculated directly by the DAK software (Schmid & Partner Engineering AG (Zürich, Switzerland) installed on a laptop connected to the VNA. For each sample of GABA concentrations, 100 repeated DRS measurements were performed. During each of the 100 repeated measurements, 167 data points were sampled. All measurements were performed in the frequency range of 200 MHz to 14 GHz. Each frequency point takes 200 μs to measure resulting in 33.4 ms to complete one frequency sweep.

## Long short-term memory artificial recurrent neural network

Long short-term memory networks (LSTM), are a special type of recurrent neural network (RNN). An RNN is a set of algorithms that are modelled after the node structures of the human brain. RNNs are widely used for natural language processing [12], as they can store both the current information and the previous information. Compared to the traditional RNN, internal mechanisms called “gates” are added to the LSTM network architecture, to regulate the flow of information. These gates decide which data points in a time sequence to keep and to forget. Keeping important information and discarding unimportant information, reduces the vanishing gradient problem. It also increases the accuracy of the predictions by reducing the information to what the algorithm deems to be relevant.

LSTM have been shown to be well-suited for classifying, processing, and making predictions with non-linear changes in time series data. Although conventionally used for time-series data, the sequential structure of the spectroscopic data also allows this architecture to be employed in learning patterns over frequencies. Permittivity data at low frequencies may be connected to the values at high frequencies, and there might be a long-term dependency between these.

## Ethical approval

The conducted research is not related to either human or animal use.

## Results

**Figure 1:**
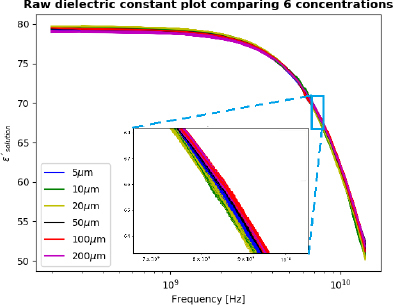
Raw dielectric constant spectra as a function of frequency for 6 GABA concentrations investigated. 100 spectra for each of the concentrations. From [13], with permission.

As shown in figure 1 there is a high degree of similarity between each of the measured permittivity spectra with different concentrations of GABA, making it dificult to discern the differences with the naked eye. This is because the contribution from the water molecules dominates the measured dielectric constant, while the contributions from the GABA concentrations are relatively small in comparison.

**Figure 2:**
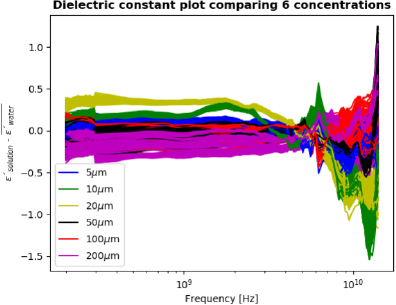
Water contribution subtracted dielectric constant spectra, as a function of frequency for 6 GABA concentrations. The average of 100 measurements of the water dielectric constant spectrum was subtracted from each of the GABA solution measurements. From [13], with permission.

Figure 2 shows the dielectric constant spectra after subtracting the water molecule contribution from the total dielectric behavior of the mixture solution. The differences between each concentration are more pronounced. For testing of the LSTM machine learning model, two datasets were used; one is the raw permittivity data from the measurements, and another is where the water contribution is subtracted from the raw data.

To assess the performance of the LSTM model, the model was used to compare the DRS data from the different concentrations, to see how accurately the model could classify different concentrations. This was done by comparing pairs of concentrations, shown in Table 1.

**Table 1:** Performance of the LSTM neural network. Raw ϵ′ data is the measured dielectric constant data of GABA solutions, whereas the subtracted ϵ′ data is the difference between the measured dielectric constant data of the GABA solutions and the average dielectric constant data of deionized water. From [13], with permission.

	Raw ϵ′ data		Subtracted ϵ′ data	
c [μm]	Test dataset Accuracy	Test dataset Loss	Test dataset Accuracy	Test dataset Loss
5 vs 10	1.0	0.00609	1.0	0.00007
10 vs 20	1.0	0.00449	1.0	0.00004
20 vs 50	1.0	0.00139	1.0	0.00009
50 vs 100	1.0	0.06400	1.0	0.00270
100 vs 200	1.0	0.08303	1.0	0.00204

In Table 1, we compare the results when using the raw dielectric data in the model to classify the GABA concentrations, to using the data where the dominating water contributions have been subtracted. Comparing the test losses for the two cases, we observed that the test losses were overall much lower when the subtracted data was used than when the raw data was used as the model input data.

**Figure 3:**
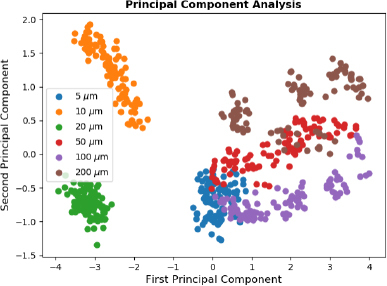
Principal component analysis on six different GABA concentrations. The X-axis shows the first component which covers 81.8% of the variance in the dataset, the Y-axis shows the second component which covers 10.4% of the variance in the dataset. From [13], with permission.

To increase the interpretability, with the goal of understanding the machine learning results better, principal component analysis (PCA) was used on the whole dataset, where all 6 concentrations were mixed randomly. PCA retains the variance in the datasets while reducing the dimensionality making it easier to visualize how separable the different GABA concentrations are from each other. Figure 3 shows the first two principal components of the dielectric constant data with water contribution subtracted. The first component covers 81.8% of the variance in the dataset, and the second component covers 10.4% of the variance in the dataset.

Figure 3 shows the clustering of the six classes with PCA, 5 μm and 10 μm are well separated from each other and from the rest of the concentrations. Whereas concentrations of 20 μm, 50 μm, 100 μm and 200 μm have more overlaps, and these overlaps make the separation between these concentrations more dificult. When comparing the PCA results from Figure 3 with the results obtained from Table 1, the test losses for the concentrations of 20 μm, 50 μm, 100 μm and 200 μm, are correspondingly higher than those of 5 μm and 10 μm GABA solutions, correlating with the degree of overlap.

To investigate whether the LSTM model could be useful for classifying any random set of GABA measurements, we used the model on the randomly mixed dataset including all measurements from all GABA concentrations measured, and the model was extended to a classification problem with six classes. Table 2 presents the results from an LSTM model that differentiates all six concentrations of GABA solutions.

**Table 2:** LSTM model results on a dataset consisting of six different GABA concentrations. “Raw data” means unprocessed dielectric constant data obtained from the GABA solution measurements. “Subtracted data” implies that the experimental dielectric constant of water (data averaged over 100 measurements) was subtracted from each of the dielectric constant data for the GABA solutions. From [13], with permission.

	Test Accuracy	Test Loss
Raw data	0.90164	0.39048
Subtracted data	0.99167	0.01064

As with the pairwise comparisons in 1, a higher test accuracy was achieved when using data where the contribution from the water molecules had been subtracted, compared to using raw data.

## Discussion

In this study, we explored a novel use of DRS to measure physiological concentrations of GABA solved in deionized water. Changes in GABA concentrations in solvent water led to changes in the dielectric properties of the solutions, that were detectable by permittivity measurements. Physiological GABA concentrations dissolved in water can be classified based on DRS measurements, through the utilization of LSTM machine learning model.

The results showed that the LSTM network model was able to classify physiological concentrations of GABA solutions with a test accuracy of 100% when performing pairwise tests. Using the LSTM model with the randomly mixed data in the test data sets yielded accuracies that were still very high, but more realistic. We acknowledge that the limited dataset used in this study probably contains sources of noise. Possible noise sources are electrical noise including environmental EMC noise, instrumentation techniques, and probe or table movement during the measurements. Also, as there was a high similarity between the different concentrations, and a large part of the measurement was dominated by the dielectric properties of the solvent water molecules, so the signal-to-noise ratio was estimated to be low.

Higher accuracies were achieved when the contribution from water was subtracted. After subtracting the contribution from the water molecules, we are left with a “cleaner” dataset that only contains contributions from the GABA molecules and residual noise. With a reduced dataset, the complexity is also reduced, making it easier for the machine learning model to extract relevant features from the data.

Training and testing with the LSTM model revealed that it is a suitable model for differentiating behind different physiological concentrations of GABA, based on DRS measurements. The LSTM model’s ability to select information not only with respect to time series, but also to keep a memory and look for patterns when sequentially going through spectroscopic data visualized as curve forms, makes it suitable to classify spectroscopic data, where small and non-linear changes occur. The lowest concentration of GABA that we were able to measure was 5 μm.

Nevertheless, the obtained results are limited to the data used, and the model trained in this work cannot be generalized for the determination of physiological concentrations of GABA without further testing. The potential of machine learning techniques for the classification of different physiological concentrations of GABA cannot be fully evaluated until the experiment is repeated with the same GABA concentration solutions several times and measurements are done over different days on different samples to assess if there are changes over time. Using data that was obtained from a single GABA solution sample (one GABA solution sample for each concentration) that was measured over a period of one day, creates a bias in the evaluation of the model. Samples of the same concentration need to be made and measured several times using independent DRS calibrations on different days to cover a wide sample and potential changes in environmental noise. In addition, considering that it is unknown whether GABA will undergo chemical changes over time when dissolved in water, new solutions should be made for each experiment day.

We acknowledge the limitations with the pure water environment of GABA used in our experiment, compared to the real-life GABA environment in the brain. The experimental setup is a very artificial laboratory setup compared to the heterogeneous and complex biological systems, such as those found in the mammalian brain. However, DRS is a simple and non-destructive method that can be used to directly assess the presence of GABA in a tissue or a sample. DRS is also a much faster measurement method compared to the methods mentioned in the introduction, providing measurements with a high temporal resolution. This work established a proof of concept that GABA physiological concentrations in water can be measured by DRS. The results from this pilot study can be used to construct new experiments to explore the possibilities of utilizing DRS measurements to detect GABA dissolved in a cell culture medium and eventually in the brain.
